# A Simple Low-Temperature Glass Bonding Process with Surface Activation by Oxygen Plasma for Micro/Nanofluidic Devices

**DOI:** 10.3390/mi11090804

**Published:** 2020-08-25

**Authors:** Koki Shoda, Minori Tanaka, Kensuke Mino, Yutaka Kazoe

**Affiliations:** Department of System Design Engineering, Faculty of Science and Technology, Keio University, 3-14-1 Hiyoshi, Kohoku, Kanagawa 223-8522, Japan; koki.shoda@tfe.sd.keio.ac.jp (K.S.); minori.tanaka@tfe.sd.keio.ac.jp (M.T.); kensuke.mino@tfe.sd.keio.ac.jp (K.M.)

**Keywords:** microfluidics, nanofluidics, fabrication, bonding, glass

## Abstract

The bonding of glass substrates is necessary when constructing micro/nanofluidic devices for sealing micro- and nanochannels. Recently, a low-temperature glass bonding method utilizing surface activation with plasma was developed to realize micro/nanofluidic devices for various applications, but it still has issues for general use. Here, we propose a simple process of low-temperature glass bonding utilizing typical facilities available in clean rooms and applied it to the fabrication of micro/nanofluidic devices made of different glasses. In the process, the substrate surface was activated with oxygen plasma, and the glass substrates were placed in contact in a class ISO 5 clean room. The pre-bonded substrates were heated for annealing. We found an optimal concentration of oxygen plasma and achieved a bonding energy of 0.33–0.48 J/m^2^ in fused-silica/fused-silica glass bonding. The process was applied to the bonding of fused-silica glass and borosilicate glass, which is generally used in optical microscopy, and revealed higher bonding energy than fused-silica/fused-silica glass bonding. An annealing temperature lower than 200 °C was necessary to avoid crack generation by thermal stress due to the different thermal properties of the glasses. A fabricated micro/nanofluidic device exhibited a pressure resistance higher than 600 kPa. This work will contribute to the advancement of micro/nanofluidics.

## 1. Introduction

The field of microfluidics has rapidly developed to realize various applications, including chemical analysis and synthesis, medical diagnosis, and tissue engineering [1,2,3]. Recently, the field has been further downscaled to nanofluidics, exploiting volumes of attoliters to femtoliters where surface effects are dominant [4]. Novel applications have been reported such as single-molecule sorting [5] and analysis [6], high-efficiency separation utilizing solid/liquid [7] and liquid/liquid phases [8], single-cell proteomics [9], and autonomous solar-light-driven fuel cells [10]. To realize these devices, micro/nano fabrication technologies are important. Recently, the direct writing technologies, such as 3D printing using polymer materials, has rapidly developed to realize easy and high-throughput production [11,12,13]. However, the fabrication of nanostructures like nanopillars and nanochannels is still difficult. Currently, polydimethylsiloxane (PDMS)-based fabrication technologies are most widely used. Microchannels can be easily fabricated by replica molding technique (soft lithography) [14]. PDMS-made devices have optical transparency suitable for detections, and surface chemical modification utilizing surface silanol groups is available. However, maintaining the shape of nanostructure is difficult due to its softness, and the material’s solubility to organic solvents and gas permeability restrict its use. On the other hand, glass-based fabrication technologies are challenging compared with others, but can achieve glass-made devices with mechanical robustness suitable for realizing nanostructures, chemical stability to organic solvents, optical transparency for detections and availability of surface chemical modification.

Fabrication methods for micro/nanofluidic devices made of glass have been reported. Micro- and nanochannels are usually fabricated on a glass substrate by wet etching and plasma etching, respectively, after the channel patterns are formed by lithography [15,16]. Surface modification of micro- and nanochannels is performed by treating with reagents [7,10] or by patterning [6,8]. The methods for bonding glass substrates are essential to construct devices by sealing microchannels and nanochannels on a substrate with another substrate. The strongest form of bonding is by thermal fusion of glass substrates at high temperatures (1080 °C in the case of fused silica, 550 °C in the case of borosilicate) [17,18,19]. Glass bonding at room temperature by surface pre-treatment with a solution of hydrofluoric acid has been reported [16,20]. Anodic bonding method at temperatures of 300 to 500 °C has been used for bonding of borosilicate glass substrates with a conductive interlayer [19,21,22]. These bonding methods are problematic for the fabrication of micro/nanofluidic devices integrating various functional materials. Most of the functional materials utilized, such as self-assembled monolayers, catalysts and modified electrodes in micro- and nanochannels, do not tolerate the extremely high temperatures needed for thermal fusion. Using hydrofluoric acid, which is a typical etchant for glass, can damage micro/nanostructures fabricated on the glass substrate. The conductive interlayer required for anodic bonding often reduces the chemical tolerance of the devices.

In order to solve these problems in bonding, recent studies have developed a method for bonding glass substrates by surface treatment with plasma and heating at low temperatures [23,24]. In this method, the glass surface is treated with oxygen plasma containing fluorine atoms for surface activation with moderate hydrophobization. After bringing the glass surfaces into contact, the substrates are heated at relatively low temperatures for annealing while pressing at 5000 N. The bonding strength was sufficient for endurance during driving fluids by pressures in micro- and nanochannels on the order of 1000 kPa. The low-temperature glass bonding method has contributed to the realization of micro/nanofluidic devices integrating various functional materials, as reported in recent studies [6,8,9,10]. In addition, detachable glass bonding with controlled bonding strength was realized by optimizing the bonding conditions [25]. Besides the methods utilizing oxygen plasma containing fluorine atoms, a sequential plasma activation process consisting of oxygen reactive ion etching plasma and nitrogen radical plasma was developed for room-temperature bonding of Si/glass and glass/glass wafers [26]. To decrease damages to the surface, a low-temperature bonding process utilizing surface activation by ultraviolet/ozone was also developed to achieve bonding of Si/Si and quartz/quartz wafers [27].

However, the low-temperature glass bonding method still has issues for general use. Adding fluorine atoms into oxygen plasma and heating the substrates while pressing with a controlled pressure requires the use of customized facilities in some cases. Furthermore, the bonding of different types of glass has not been achieved. Previous studies have verified the low-temperature bonding of fused-silica glass substrates [23,24,25,28], which is identical to the glass material used for semiconductor fabrication. However, since microscope objective lenses are optimally designed for borosilicate cover glass (refractive index: 1.52), the use of micro/nanofluidic devices made of fused-silica glass (refractive index: 1.46) results in reduced spatial resolution during microscopic observation, as reported in a previous study [29]. Thus, the bonding of fused-silica and borosilicate glass substrates is important for combining micro/nanofluidic devices and microscopic optical detection.

In the present study, we developed a low-temperature glass bonding process utilizing typical facilities available in clean rooms, and applied the process to fabrication of micro/nanofluidic devices. A bonding process with surface activation by oxygen plasma and without pressing substrates during the annealing was proposed. The oxygen plasma and heating temperature conditions were investigated, and the bonding of fused-silica glass substrates was verified by measuring the bonding energy. The verified bonding process was applied to the bonding of fused-silica and borosilicate glass substrates. The bonding energy of fused-silica/borosilicate glass substrates was evaluated. In addition, the pressure resistance of a micro/nanofluidic device made of fused-silica and borosilicate glass was evaluated by investigating liquid leakage from a nanochannel. This work provides a simple and easy low-temperature glass bonding process for the fabrication of micro/nanofluidic devices.

## 2. Materials and Methods

### 2.1. Materials

Fused-silica glass substrates with thicknesses of 0.25 and 0.70 mm (70 mm × 30 mm, VIO-SILSX, Shin-Etsu Quartz Co., Ltd., Tokyo, Japan) and borosilicate glass substrates with thicknesses of 0.17 and 0.25 mm (70 mm × 30 mm, Matsunami Glass Ind., Ltd., Osaka, Japan) were prepared. The 0.17-mm borosilicate glass is used as a standard cover glass for microscopy. The Young’s modulus of fused-silica glass and borosilicate glass was 7.38 × 10^10^ Pa and 7.29 × 10^10^ Pa, respectively, according to the manufactures. The surface roughness of glass substrates was less than 0.3 nm.

For the confirmation of liquid leakage from nanochannels, a fluorescent aqueous solution, including 0.1-mmol/L fluorescein and phosphate-buffered saline was prepared.

### 2.2. Fabrication of Micro/Nanofluidic Devices

Micro/nanofluidic devices were fabricated by the top-down fabrication of micro- and nanochannels on glass substrates, based on reported methods [8,30,31]. Nanochannels with sizes of 100 to 1000 nm were fabricated by electron beam lithography and dry etching. In the electron beam lithography process (F5112+VD01, Advantest Corp., Tokyo, Japan), ZEP-520A (Zeon Corp., Tokyo, Japan) was used as the resist. After forming the channel pattern, dry etching (NLD-570, ULVAC Co., Ltd., Kanagawa, Japan) was performed using a mixture of gaseous CHF_3_ and SF_6_. To fabricate nanochannels of 10 to 100-μm width and 100 to 1000-nm depth, the channel pattern was formed by photolithography using THB-111N (JSR Corp., Tokyo, Japan) as a photoresist. On the other hand, microchannels were fabricated by photolithography and dry etching. KMPR^®^1035 (Kayaku Co., Ltd., Tokyo, Japan) was used as a photoresist for micrometer-scale dry etching using a mixture of gaseous C_3_F_8_, CHF_3_ and Ar. Inlet holes with a diameter of 0.7 mm were produced on the substrate using a diamond-coated drill. After fabricating the channels and inlet holes, the device was constructed by the low-temperature bonding of the glass substrates as described in the following section.

### 2.3. Low-Temperature-Bonding of Glass Substrates

**Figure 1:**
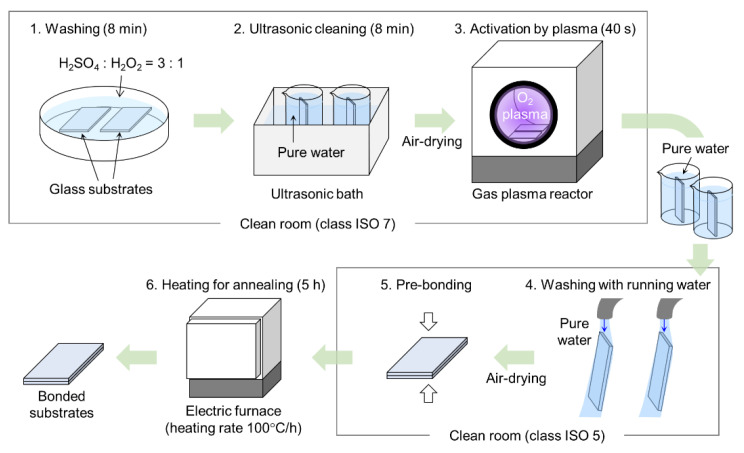
Schematic diagram of low-temperature bonding process for glass substrates utilizing typical facilities in clean rooms.

In the present study, we propose a process of low-temperature glass bonding utilizing typical facilities available in clean rooms. Figure 1 illustrates schematics of the bonding process. In a class ISO 7 clean room, washing and activation of the surfaces of the glass substrates were performed. In the washing step, the substrates were immersed in a three-to-one mixture of sulfuric acid and hydrogen peroxide for 8 min. After cleaning the substrates by ultrasonication for 8 min and air-drying, the substrate surface was activated by irradiation with an oxygen plasma for 40 s at a power of 200 W, utilizing a gas plasma reactor (PR510, Yamato Scientific Co., Ltd., Tokyo, Japan). Then, the substrates were immersed in pure water to prevent contamination by particles in the atmosphere and transported to a class ISO 5 clean room. After washing the substrates with running pure water and air-drying, the substrates were brought into contact for pre-bonding. In this step, air often remained in the bonding interface and generate voids. In such case, air in the bonding interface was ejected by manual pressure. Finally, the pre-bonded substrates were heated for annealing in an electric furnace (FO200, Yamato Scientific Co., Ltd., Tokyo, Japan) at a constant temperature for 5 h with a heating rate of 100 °C/h.

To optimize the proposed bonding process, the effects of the pressure in the plasma reaction chamber and the heating temperature used for annealing on the bonding strength were investigated. The pressure in the reaction chamber was set to 40, 51 and 60 Pa by providing oxygen at a flow rate of 45, 70 and 100 mL/min, respectively, with evacuation by a vacuum pump. The heating temperature was set at 20 (room temperature), 50, 110, 200, 300 and 400 °C.

### 2.4. Evaluation of Bonding Strength

**Figure 2:**
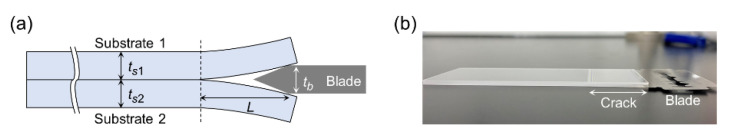
(**a**) Principle of crack-opening test and (**b**) photograph of bonded glass substrates with a blade inserted into the bonding interface.

The bonding strength of glass substrates was measured by a crack-opening test [32], which is a standard method for evaluating bonding. A stainless-steel blade with a thickness of 0.1 mm (Hi-Stainless, Feather Safety Razor Co., Ltd., Osaka, Japan) was used. As shown in Figure 2a, the stainless blade was inserted at the bonding interface, and the crack propagation length, *L*, was measured. The bonding energy, *γ*, between substrate 1 and substrate 2 is given by:

(1) $$\gamma =\frac{3t_{b}^{2}E_{s1}t_{s1}^{3}E_{s2}t_{s2}^{3}}{16L^{4}\left(E_{s1}t_{s1}^{3}+E_{s2}t_{s2}^{3}\right)}\;,$$

where *t_b_* is the thickness of the blade, *t_s_* is the thickness of the glass substrate, and *E_s_* is Young’s modulus [28]. As shown in Figure 2b, the blade was inserted into the bonding interface between glass substrates, and the crack propagation length was measured.

The pressure resistance of the micro/nanofluidic device was examined by observing the leakage of a fluorescein solution from a nanochannel. The fluorescence was observed by an inverted fluorescence microscope (IX71, Olympus Corp., Tokyo, Japan) combined with an objective lens (20×, NA = 0.75), a 1.6× magnification lens, and a complementary metal-oxide-semiconductor (CMOS) camera (C11440-36U, Hamamatsu Photonics K. K., Hamamatsu, Japan). The pixel size of the CMOS camera was 5.86 μm, and the exposure time was set at 70 ms. The fluorescein solution was injected into the device by air pressure generated by a pressure controller (Institute of Microchemical Technology Co., Ltd., Kanagawa, Japan).

## 3. Results and Discussion

### 3.1. Bonding of Fused-Silica/Fused-Silica Glass Substrates

**Figure 3:**
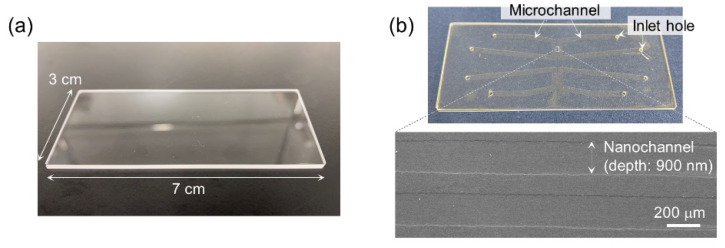
Low-temperature bonding of fused-silica glass substrates. (**a**) Photograph of bonded fused-silica glass substrates. (**b**) Photograph of a micro/nanofluidic device fabricated by bonding of a fused-silica glass substrates with microchannels and nanochannels.

Figure 3 shows photographs of fused-silica glass substrates bonded by low-temperature bonding according to the process shown in Figure 1. The pressure in the plasma reaction chamber and the annealing temperature were set at 51 Pa and 400 °C, respectively. As shown in Figure 3a, the glass substrates were bonded without residual voids at the interface between the substrates. The bonding process was applied to the fabrication of a micro/nanofluidic device made of fused-silica glass. As shown in Figure 3b, the device included nanochannels of 400 μm wide and 900 nm deep, which were interfaced with microchannels of 1.7 mm wide and 30 μm deep. As well as the bonding of glass substrates without any fabrication, the bonding of glass substrates with fabricated micro- and nanochannels was realized. We note that, among the bonding process (Figure 1), when we conducted processes of “washing with running water” and “pre-bonding” in a class ISO 7 clean room, the substrate surface was contaminated by a large number of particles from the atmosphere, and the bonding failed. This result suggests the importance of using a class ISO 5 clean room during these processes to achieve successful bonding.

**Figure 4:**
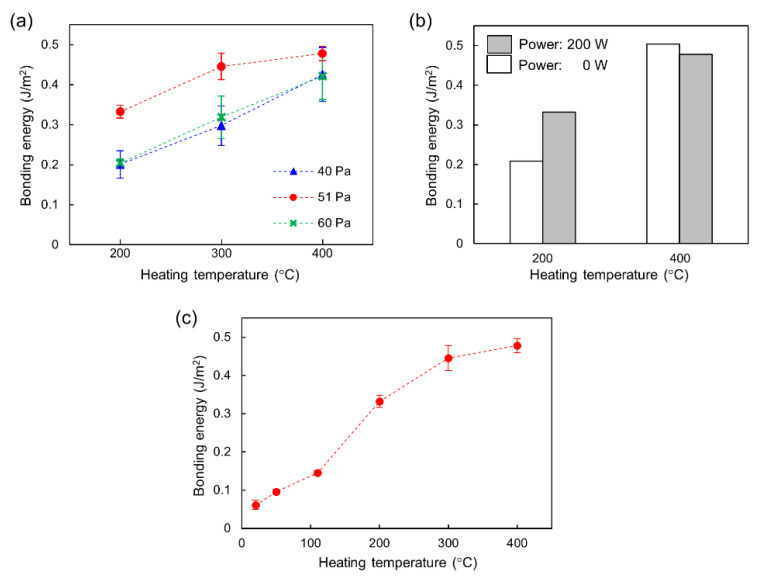
Bonding energy measured by a crack-opening test. (**a**) Bonding energy as a function of heating temperature at different pressures in a reaction chamber, indicating the concentration of oxygen plasma. (**b**) Comparison of bonding energy for plasma power of 0 and 200 W. (**c**) Bonding energy as function of heating temperature at a pressure in reaction chamber of 51 Pa.

In order to evaluate the bonding strength of the fused-silica glass substrates, we performed a crack-opening test. Figure 4a shows the bonding energy as a function of the heating temperature for a different plasma reaction chamber pressure conditions. For each condition, we repeated experiments at least four times and obtained standard deviation. The bonding energy increased with increasing heating temperature. We found that a moderate concentration of oxygen plasma with the pressure of 51 Pa could achieve high bonding energies ranging from 0.33 to 0.48 J/m^2^ with heating temperatures ranging from 200 to 400 °C, which are comparable to that corresponding to a pressure resistance on the order of 100 kPa, as reported previously [23,24,25]. Therefore, in case of bonding fused-silica glass substrates, we verified the process of low-temperature glass bonding utilizing typical facilities available in clean rooms.

We also investigated the effects of surface activation by oxygen plasma on the bonding energy. Figure 4b shows a comparison of the bonding energy at different plasma powers (0 and 200 W). The pressure in the reaction chamber was set at 51 Pa. At a heating temperature of 200 °C, the bonding energy with surface activation for a power of 200 W increased 1.7 times compared to that without surface activation (power: 0 W). At a heating temperature of 400 °C, the bonding energies at powers of 0 and 200 W became comparable. The results indicate that, for lower heating temperatures, the bonding strength is increased by activating the surface.

The results were evaluated to consider the mechanism involved in low-temperature glass bonding, which is not yet fully understood. Recently, Wang et al. [28] experimentally investigated surface properties during bonding by contact angle measurement, atomic force microscopy, Raman scattering spectrometry and X-ray photoelectron spectroscopy, and proposed the following bonding mechanism. Upon activation by oxygen plasma, the oxygen free radicals break the covalent bonds of siloxane (Si-O-Si) on the glass surface and produce dangling Si-O- groups by the reaction, Si-O-Si + O* → Si- + Si-O-. After activation, water molecules are adsorbed on the surface to form silanol groups (Si-OH) by the reaction, Si- + Si-O- + H_2_O → Si-OH + Si-OH, and the hydrophilicity of the surface increases. During the annealing after the pre-bonding, a dehydration reaction of silanol groups between the pair of substrates occurs to form covalent bonds of siloxane, according to Si-OH + Si-OH → Si-O-Si + H_2_O. As a result, the bonding of the substrates is achieved. Xu et al. [23,24] and Ohta et al. [25] indicated that an increase in bonding energy could be achieved by adding fluorine plasma in the activation process. They suggested that moderate hydrophobicity of the surface is necessary for the ejection of residual water molecules from the bonding interface to enhance the bonding. The results obtained in the present study basically support the models considered in these previous studies, and provide new insights to understand the bonding mechanism. Namely, finding the optimal concentration of oxygen plasma as shown in Figure 4a suggests that strong bonding can be achieved by an optimal balance between the amount of hydrophilic silanol groups (Si-OH) to produce bonding and that of hydrophobic siloxane (Si-O-Si) to eject water molecules from the bonding interface. In addition, the result shown in Figure 4b suggests that the effect of increased Si-OH due to surface activation on the bonding strength is significant at 200 °C, while it becomes insignificant at 400 °C because the dehydration reaction of Si-OH for bonding is sufficiently accelerated at temperatures higher than 200 °C, as reported in previous studies on the surface chemistry of silica [33].

At the optimized pressure in reaction chamber of 51 Pa, we further investigated the heating temperature for annealing. Figure 4c shows the results of a crack-opening test to measure the bonding energy as a function of heating temperature ranging from 20 (room temperature) to 400 °C. The bonding energy significantly decreased when the heating temperature decreased to 110 °C, probably because of the insufficient dehydration reaction of silanol groups. Therefore, in case of bonding fused-silica glass substrates, the heating temperature higher than 200 °C is necessary to obtain a sufficient bonding energy for nanofluidic devices, based on previous studies [23].

### 3.2. Bonding of Fused-Silica/Borosilicate Glass Subsrates

**Figure 5:**
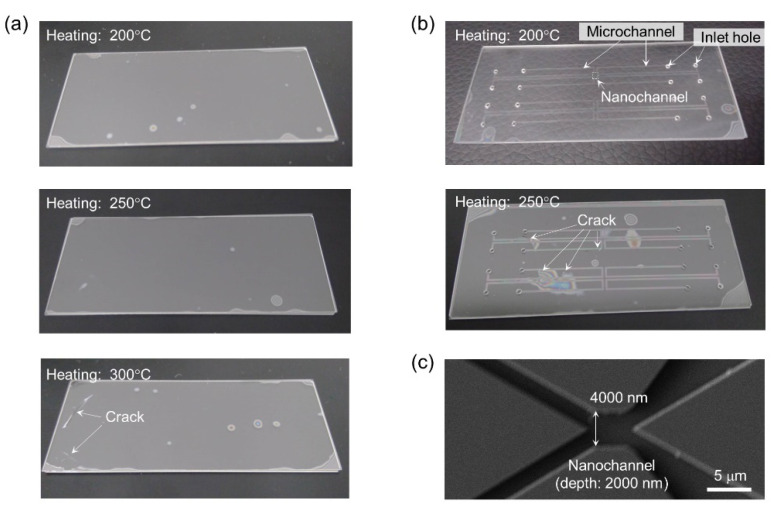
Low-temperature bonding of fused-silica glass substrate (thickness: 0.7 mm) and borosilicate glass substrate (thickness: 0.17 mm). (**a**) Photograph of bonded fused-silica and borosilicate glass substrates. (**b**) Photograph of micro/nanofluidic device fabricated by the bonding of fused-silica substrate with micro- and nanochannels and a borosilicate glass substrate. (**c**) X-shaped nanochannel of 4000 nm wide and 2000 nm deep fabricated in a boxed area indicated on the photograph of micro/nanofluidic device (Figure 4b).

Based on the results shown in Figure 4, the low-temperature glass bonding method with an optimized pressure of 51 Pa was used to bond fused-silica and borosilicate glass substrates. Figure 5 shows the results of bonding at different heating temperatures. The 0.17-mm borosilicate glass substrate, which is a standard cover glass for microscopy, was used. In the case of substrates without any fabrication (Figure 5a), the bonding was successful at heating temperatures of 200 and 250 °C. However, at a heating temperature of 300 °C, cracks were generated owing to the thermal stress caused by the order of magnitude difference in thermal expansion coefficients between fused-silica (0.5 × 10^−6^ K^−1^) and borosilicate (7.2 × 10^−6^ K^−1^). On the other hand, in the case of substrates with micro- and nanochannels (Figure 5b), cracks were generated even at a temperature of 250 °C, probably because of stress concentration at the places where the channels were fabricated. At a temperature of 200 °C, we succeeded in fabricating a micro/nanofluidic device containing microchannels (width: 500 μm, depth: 10 μm) and nanochannels, as shown in Figure 5c. The results suggest that the fabrication of micro- and nanochannels affects an appropriate heating temperature for bonding different glasses due to the concentration of thermal stress. Therefore, to fabricate micro/nanofluidic devices by bonding fused-silica and borosilicate glass substrates, using a heating temperature lower than 200 °C is important to avoid crack generation due to the thermal stress.

**Figure 6:**
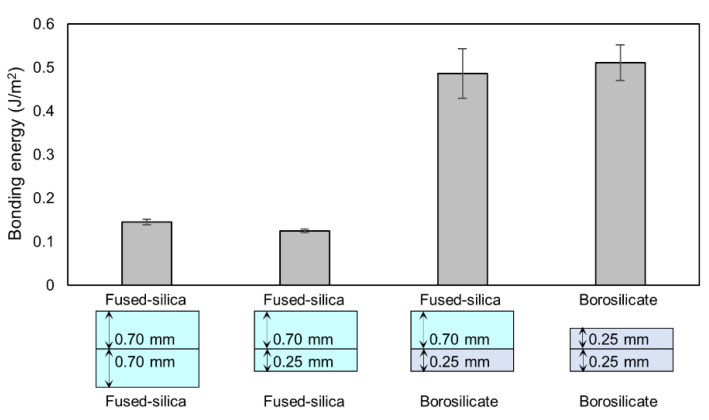
Bonding energies of fused-silica/fused-silica, fused-silica/borosilicate and borosilicate/borosilicate glass substrates at a heating temperature of 110 °C, measured by crack-opening test.

Since the borosilicate glass substrate with a 0.17-mm thickness was too fragile to perform a crack-opening test, we instead used the borosilicate glass substrate with a 0.25-mm thickness to examine the bonding energy at the heating temperature lower than 200 °C. The bonding energies of fused-silica/fused silica, fused-silica/borosilicate and borosilicate/borosilicate glass substrates at a heating temperature of 110 °C are shown in Figure 6. To confirm the effect of the substrate thickness, a fused-silica glass substrate with a thickness of 0.25 mm was also used. The results suggest that the bonding energy is independent on the substrate thickness but dependent on the glass material. When the borosilicate glass was used, the bonding energy was more than three times higher than that in case of fused-silica/fused-silica glass bonding. The bonding energies of fused-silica/borosilicate and borosilicate/borosilicate glass substrates were around 0.5 J/m^2^ at a heating temperature of 110 °C, which is sufficient for nanofluidic devices based on previous studies [23]. We note that, at a heating temperature of 200 °C, we could not insert the blade without breaking the glass because of further increased bonding energy. Therefore, in case of bonding fused-silica/borosilicate glass substrates, the heating temperature lower than 200 °C is an appropriate condition for nanofluidic devices. Several reasons for the increased bonding energy by using borosilicate glass can be considered, such as a different density of silanol group on the surface and B_2_O_3_ or other cations included in borosilicate glass. The previous study revealed that washing the substrate with calcium acetate solution can enhance the low-temperature bonding of borosilicate/borosilicate glasses [34]. Strong borosilicate/borosilicate glass bonding without annealing was also achieved by washing substrates with sulfuric acid and high-flow-rate tap water [35]. Although the mechanism is still not understood, the higher bonding energy of borosilicate glass compared with that of fused-silica glass may support the validity of these previous studies on the low-temperature bonding of borosilicate glass.

**Figure 7:**
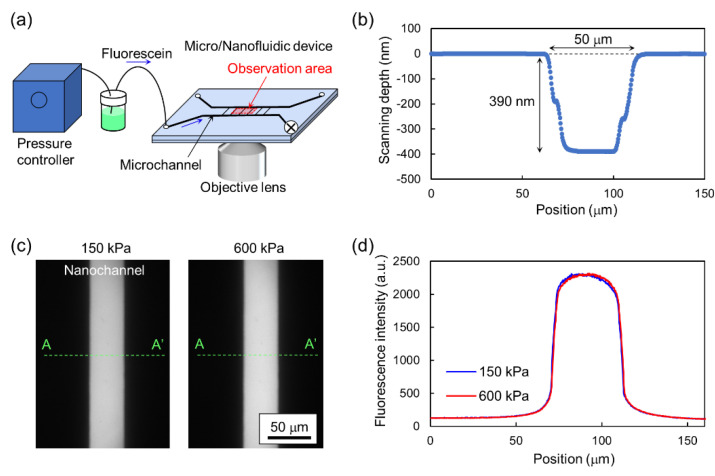
Examination of the pressure resistance of a micro/nanofluidic device by fluorescence microscopy. (**a**) Experimental setup. (**b**) Cross-sectional profile of a nanochannel measured by stylus surface profiler. (**c**) Fluorescence images of nanochannel where fluorescein solution was injected by external pressure. (**d**) Profiles of fluorescent intensity in transverse direction of nanochannel (A-A′).

Finally, we examined the pressure resistance of a micro/nanofluidic device by fluorescence microscopy, which previous studies have employed to evaluate the bonding strength [23,24,25]. Figure 7a shows the experimental setup. The device was fabricated by bonding a fused-silica glass substrate with microchannels (width: 500 μm, depth: 10 μm) and nanochannels (width: 50 μm, depth: 390 nm), as shown in Figure 7b, and a 0.17-mm borosilicate glass substrate without any patterning. In the bonding process, the heating temperature was set at 200 °C. A fluorescein solution was injected into the nanochannels via the microchannel. Since the pressure loss in microchannels is negligibly small compared to that in nanochannels, an external pressure applied to the device generated by the pressure controller was regarded as that applied to the nanochannels. Fluorescence images of the nanochannel at applied pressures of 150 and 600 kPa were obtained, as shown in Figure 7c.

Figure 7d shows profiles of the fluorescence intensity of the nanochannel. The area of the nanochannel shows bright fluorescence, while that outside the nanochannel is dark, with an image intensity similar to the background. In addition, the image intensity outside the nanochannel was constant even when the applied pressure was increased from 150 to 600 kPa. These results indicate that no detectable leakage of the fluorescein solution from the nanochannel occurred and bonding of the glass substrates was achieved. Therefore, it is concluded that the proposed bonding process was successfully applied to the micro/nanofluidic device made of fused-silica and borosilicate glass, and the device had a pressure resistance higher than 600 kPa. Micro/nanofluidic devices fabricated by the proposed bonding process can be used for various microfluidic applications [1,2], nanofluidic applications with pressure-driven flows generated by high external pressures on the order of 100 kPa, such as single-molecule immunoassays [6], femtoliter solvent extraction [8], and single-cell target proteomics [9], and other nanofluidic applications such as electrophoretic single-molecule sorting [5] and miniaturized fuel cells [10]. In addition, the construction of devices made of fused-silica and borosilicate glass enables a combination of micro/nanofluidics and advanced optical microscopy such as super-resolution microscopy [36,37] without a reduction in performance.

## 4. Conclusions

We developed a simple process for low-temperature glass bonding utilizing typical facilities available in clean rooms and applied it to the fabrication of micro/nanofluidic devices by the bonding of fused-silica/fused-silica glass substrates and fused-silica/borosilicate glass substrates. The proposed bonding process was verified, and the bonding energy for fused-silica/fused-silica substrates at the annealing temperature of 200–400 °C was 0.33–0.48 J/m^2^, which is comparable to that reported in previous studies. We found that a moderate concentration of oxygen plasma with a pressure of 51 Pa for activating the surface of glass substrates could achieve strong bonding. The enhancement of bonding strength by activating the surface with oxygen plasma was significant for annealing at 200 °C, while it was insignificant at 400 °C. These results provide new insights to help understanding the bonding mechanism. The optimized bonding process was applied to the fabrication of micro/nanofluidic devices made of a fused-silica glass substrate and a borosilicate glass substrate (0.17 mm thick), which is generally used for microscopic optical measurements. In the bonding process, using an annealing temperature lower than 200 °C was important to avoid crack generation by thermal stress due to the difference in thermal expansion coefficients between fused silica and borosilicate. At an annealing temperature of 110 °C, the bonding energy was around 0.5 J/cm^2^ and three times higher than that of fused-silica/fused silica glass bonding. The results suggest that the bonding energy depends on the glass material and increases in case of using borosilicate glass. We succeeded in device fabrication, and the fabricated micro/nanofluidic device had a pressure resistance higher than 600 kPa, which is sufficient for various micro/nanofluidic applications. In previous studies [23,24,25], conditions of low-temperature bonding for fused-silica/fused-silica glass substrates utilizing oxygen plasma with fluorine atoms were investigated to achieve bonding energy of 0.45–1.00 J/m^2^, which corresponds to pressure resistance of nanochannels on the order of 100–1000 kPa. In the present study, we developed conditions of low-temperature glass bonding utilizing oxygen plasma without fluorine atoms and verified bonding of fused-silica/fused-silica and fused-silica/borosilicate glass substrates. Bonding energy in similar order to that by previous studies was achieved. The results provide a simple and easy process of low-temperature glass bonding for the fabrication of micro/nanofluidic devices, enable the use of high-resolution optical microscopy designed for borosilicate cover glass, and will greatly contribute to advancements in micro/nanofluidics.
